# The complete mitochondrial genome of Fea’s muntjac (*Muntiacus feae* Thomas and Doria, 1889) with phylogenetic analysis^†^

**DOI:** 10.1080/23802359.2018.1507634

**Published:** 2018-10-30

**Authors:** Suttikarn Srisodsuk, Prateep Duengkae, Urarikha Kongprom, Boripat Siriaroonrat, Sutee Duangjai

**Affiliations:** a Department of Forest Biology, Faculty of Forestry, Kasetsart University, Bangkok, Thailand;; b Khao Kheow Open Zoo, Zoological Park Organization, Chonburi, Thailand;; c Bureau of conservation and research, Zoological Park Organization, Bangkok, Thailand

**Keywords:** *Muntiacus feae*, Fea’s muntjac, mitochondrial genome

## Abstract

The complete mitochondrial genome (mitogenome) of the rare Fea’s muntjac (*Muntiacus feae*) was sequenced (GenBank accession nos. MG857662–MG857664). The mitogenome was found to be 16,355 bp in length with base compositions of 33.16% A, 24.59% C, 13.46% G, and 28.78% T and a GC content of 38.06%. The genome is comprised of 13 protein-coding genes (PCGs), 22 transfer RNA genes, 2 ribosomal RNA genes, and a control region (D-loop). Phylogenetic analysis revealed that Fea’s muntjac is more closely related to Black muntjac (*M. cronifrons*) than to Red muntjac (*M. muntjak*). These data will be useful for further studies on the genetic diversity and molecular phylogenetic relationship of the genus *Muntiacus*.

Fea’s muntjac (*Muntiacus feae* Thomas and Doria, 1889) is a rare species found only in the Tenasserim Mountains, southern and western Thailand, and parts of southern Myanmar (Lekagul and Mcneely 1977). It is categorized as one of the 15 reserved wild animals of Thailand. However, no mitogenome information for Fea’s muntjac was available until now, and its genetic relationships with other species are not well known. Therefore, we assembled and characterized the complete mitogenome of Fea’s muntjac and clarified its relationships with other species of the genus *Muntiacus* using mitogenome sequences.

Blood was collected from three Fea’s muntjac individuals from the Khao Kheow Open Zoo, Chon Buri Province Thailand. Genomic DNA was extracted from the blood samples using the Favor™ Blood Genomic DNA Extraction Mini Kit (Favorgen Biotech Corp., Pingtung, Taiwan). The complete Fea’s muntjac mitogenome was sequenced by amplifying using six long overlapping polymerase chain reaction fragments of the mitogenome, which were sequenced by the primer walking method using sequencing primers targeting the flanking sequences. The DNA sequences of each individual were assembled using AutoAssembler version 2.1.1 (Applied Biosystems, Foster City, CA). The assembled mitogenome was annotated using the MITOS WebServer (Bernt et al. 2013), Dual Organellar Genome Annotator (Wyman et al. 2004), and MitoFish (Iwasaki et al. 2013). All transfer RNA (tRNA) genes were further confirmed using the tRNAscan-SE Search Server (Lowe and Chan 2016).

The complete mitogenome of Fea’s muntjac was found to be 16,355 bp in length and was deposited in GenBank under accession nos. MG857662–MG857664. The genomes of each individual were identical, consisting of 13 protein-coding genes (PCGs), 22 tRNA genes, 2 ribosomal RNA (rRNA) genes, and a control region (D-loop). The overall base composition of the heavy strand is 33.16% A, 24.59% C, 13.46% G, and 28.78% T, with a GC content of 38.06%. Most mitochondrial genes are encoded on the heavy strand except for *ND6* and eight tRNA genes (*tRNA^Gln^*, *tRNA^Ala^*, *tRNA^Asn^*, *tRNA^Cys^*, *tRNA^Tyr^*, *tRNA^Ser^*, *tRNA^Glu^*, and *tRNA^Pro^*), which are encoded on the light strand. Most of the 13 PCGs begin with the common initiation codon, ATG, whereas *ND2*, *ND3*, and *ND5* begin with ATA. As the stop codon, 8 of the 13 PCGs end with TAA, *CytB* ends with AGA, and *ND2* ends with TAG. The other three PCGs (*COIII*, *ND3*, and *ND4*) end with T- as an incomplete stop codon. The lengths of the *12S rRNA* and *16S rRNA* genes are 956 and 1568 bp, respectively. The lengths of the 22 tRNA genes ranged from 60 bp (*tRNA^Ser^*) to 75 bp (*tRNA^Leu^*), and the inferred secondary structures of all tRNAs conform to the characteristic structural features of mitochondrial tRNAs. The control region (D-loop) is 924 bp and located between the *tRNA^Pro^* and *tRNA^Phe^* genes.

To elucidate the mitogenome of *M. feae* further and to investigate the evolution of the genus *Muntiacus*, complete mitochondrial DNA (mtDNA) sequences from 12 samples representing six species of *Muntiacus* and the outgroup *Elaphodus cephalophus* were used for phylogenetic analyses. Phylogenetic analyses were performed using both neighbour joining (NJ) and Bayesian inference. NJ analyses were performed using PAUP* (Swofford 2003), and Bayesian inference was performed using MrBayes version 3.2 (Ronquist et al. 2012). The tree topologies based on the complete mtDNA sequences in this study were identical and statistically supported by high bootstrap and posterior probability values. These results indicate that Fea’s muntjac is more closely related to Black muntjac (*M. cronifrons*) than to Red muntjac (*M. muntjak*). Only the majority-rule consensus tree derived from Bayesian inference is shown in Figure 1.

**Figure 1.**
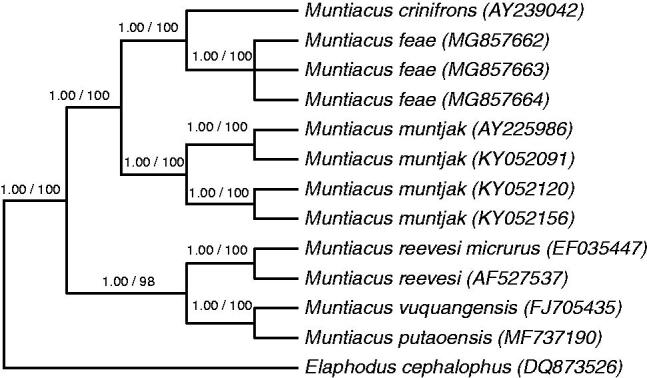
Bayesian inference phylogenetic tree of the genus *Muntiacus* based on complete mtDNA sequences. GenBank accession numbers are shown in parentheses. Numbers above the branches are the posterior probabilities and NJ bootstrap support, respectively.
